# Avoidance Behavior in Chinhai Spiny Newt Larvae: Responses to Visual and Chemical Cues from a Novel Predator

**DOI:** 10.3390/ani16020261

**Published:** 2026-01-15

**Authors:** Shiyan Feng, Wei Li, Di An, Zhiya Ma, Zhenhua Luo, Aichun Xu

**Affiliations:** 1 College of Life Sciences, China Jiliang University, Hangzhou 310018, China; fsy0714@cjlu.edu.cn (S.F.); vivia_512@outlook.com (W.L.); echoannnndy@gmail.com (D.A.); miya_ma_888@163.com (Z.M.); 2 Institute of Evolution and Ecology, School of Life Sciences, Central China Normal University, Wuhan 430079, China

**Keywords:** *Echinotriton chinhaiensis*, novel potential predator, anti-predator behavior, visual cues, chemical cues, caudate amphibian

## Abstract

Effective recognition of potential threats is crucial for survival in aquatic habitats, especially for amphibian larvae. As a critically endangered species, understanding how the Chinhai spiny newt (*Echinotriton chinhaiensis*) larvae recognize novel predators provides key scientific support for developing targeted conservation strategies. Using the American bullfrog (*Lithobates catesbeiana*) as a representative predator, we examined larval responses by presenting isolated visual or chemical cues, as well as visual cues from predators of differing body sizes. We measured larval avoidance and activity. Results showed that with only visual cues, larvae quickly avoided the bullfrog and significantly reduced their activity compared to controls. With only chemical cues, activity decreased significantly, but avoidance behavior did not. When both large and small bullfrogs were present, larvae avoided the larger individual significantly more. These findings demonstrate that *E. chinhaiensis* larvae can use visual or chemical cues to detect novel potential predators and assess risk based on size to guide their avoidance behavior. This study provides key empirical data for understanding anti-predator responses in endangered caudate amphibians and informs conservation strategies against potential threats.

## Simple Summary

This study investigates how the larvae of the Chinhai spiny newt recognize and respond to the novel predator, so we used the bullfrog as a representative predator in the experiment. Experiments showed that when only visual cues were provided, the larvae quickly recognized the bullfrog and actively moved away, with a significant reduction in activity level. When only chemical cues were provided, tadpole activity decreased, but no significant avoidance behavior was observed. When presented with both large and small bullfrogs simultaneously, the larvae showed a stronger tendency to avoid the larger bullfrog. The results indicate that the larvae can detect predation threats via both visual and chemical cues, assess risk according to predator body size, and implement appropriate avoidance strategies.

## 1. Introduction

When facing native predators, native species typically develop effective recognition mechanisms and adaptive anti-predator strategies through long-term co-evolution, such as cluster behavior [1], avoidance behavior, reduced activity level [2], etc., thereby reducing predation risk. However, native species often lack a shared evolutionary history with novel potential predators which can prevent them from recognizing and responding to such novel threats effectively or in a timely manner—a phenomenon known as prey naivety [3]. This failure in predator recognition frequently contributes to population declines among native species [4,5]. Therefore, figuring out how native prey recognize predators, especially novel predators and how they behave in response is a core prerequisite for creating conservation strategies for endangered amphibians.

In aquatic ecosystems, visual cues and chemical cues are the main ways for larvae to recognize predators. Visual cues refer to the direct observation of predators. They have the characteristics of high spatial reliability and fast transmission speed. In habitats with simple environmental structures and good visibility, visual cues are very important. Chemical cues, consisting of substances secreted or metabolically released by predators into the environment, can diffuse through water to convey information. Even in visually limited conditions, these cues remain effective in signaling the presence of threats. The perception of chemical cues by amphibian larvae has been widely documented [6]. For instance, when cane toad (*Rhinella marina*) tadpoles are exposed to chemical signals from injured conspecifics, they exhibit reduced activity, demonstrating that such chemical information functions as an alarm signal [7]. Similarly, two-lined salamander (*Eurycea bislineata*) larvae utilize chemical cues to detect the presence of carnivorous predators such as fish [8]. Chemical cues can signal the presence of a predator, whereas visual cues enable prey to precisely adjust their responses according to varying levels of threat [9,10]. Currently, research on the role of the two types of cues in risk assessment has mostly focused on groups such as fish [9] and frogs [11]. In contrast, we know very little about the innate recognition abilities of larvae in caudate amphibian, especially among rare and endangered species with restricted geographical distributions and limited historical exposure to potential predators.

The Chinhai spiny newt (*Echinotriton chinhaiensis*) is one of the most ancient amphibian species that belongs to the order Urodela and family Salamandridae Because of its rarity of natural populations, *E. chinhaiensis* is listed as a “Critically Endangered” species on the IUCN Red List of Threatened Species. Currently, it is only distributed in several narrow areas of Ningbo City, Zhejiang Province [12]. In addition to habitat limitations, predation is also a key factor contributing to its low survival rate and small population size [13]. The early development of the Chinhai spiny newt lasts about 120 days, from fertilization to the completion of metamorphosis. Among this period, its aquatic life stage takes approximately 58–88 days [14], accounting for more than half of the total development time. The larval stage of the Chinhai spiny newt is a high-risk period in its life cycle, during which it is highly vulnerable to attacks by aquatic predators. The species composition in its natural breeding pools is often complex and susceptible to invasion by non-native species, further exacerbating the survival pressure faced by the larvae during their aquatic development phase. At present, the extent to which the Chinhai spiny newt can recognize novel potential predators remains unclear. However, given the short co-evolutionary history as a “predator–prey” system, the larvae of the Chinhai spiny newt are most likely unable to quickly identify novel species and respond in a timely manner.

The bullfrog (*Lithobates catesbeiana*) is an extremely dangerous invasive alien species. It is listed as one of the 100 worst invasive species globally by the International Union for Conservation of Nature (IUCN) [15]. With high reproductive capacity and no natural predators to control its population, this species has spread rapidly, posing a serious threat to native amphibians, fish, and the stability of aquatic ecosystems [16,17]. At present, the bullfrog is classified with an invasion risk level of 2 [18] and has a widespread distribution across China, having established naturalized populations in provinces and regions such as Zhejiang, Sichuan, and Yunnan [19,20]. The bullfrog’s large size and frequent individual movements provide strong visual stimuli [21], while its metabolites and certain skin secretions, which are soluble in water, can serve as chemical cues indicating its presence [22]. Although the bullfrog has not yet established a stable wild population within the conservation area, it has been introduced into the surrounding regions through local market and restaurant trade. The larvae of the Chinhai spiny newt likely have no evolutionary history of contact with bullfrogs. As a newly emerged and ecologically impactful alien species, the bullfrog now poses a tangible threat to their survival.

This study aims to investigate the mechanisms underlying predator recognition in the Chinhai spiny newt larvae, with a focus on the roles of visual and chemical cues in detecting novel potential predators. Therefore, in this study, we used the bullfrog as a model predator. Through controlled laboratory experiments, we separately presented visual and chemical cues to analyze the responses of Chinhai spiny newt larvae to the predator. In addition, visual cues from the same predators with different body sizes were provided to compare differences in the larvae’s responses. Our study aims to address the following three questions: (1) Do Chinhai spiny newt larvae exhibit avoidance behavior when presented with only visual or only chemical cues from the predator? (2) What are the characteristics of these avoidance behaviors, and how do they affect the larvae’s activity levels? (3) Can the larvae assess predation risk based on the body size of predators and adopt appropriate avoidance behaviors?? We predict that: (1) under visual-only conditions, Chinhai spiny newt larvae will move away from the predator and reduce their activity level; (2) under chemical-only conditions, they will also avoid the predator-derived chemical cues and decrease their activity; (3) the larvae can use visual information to assess predator body size and associated risk and would preferentially avoid the larger predator.

## 2. Materials and Methods

### 2.1. Animal Collection and Maintenance

**Figure 1:**
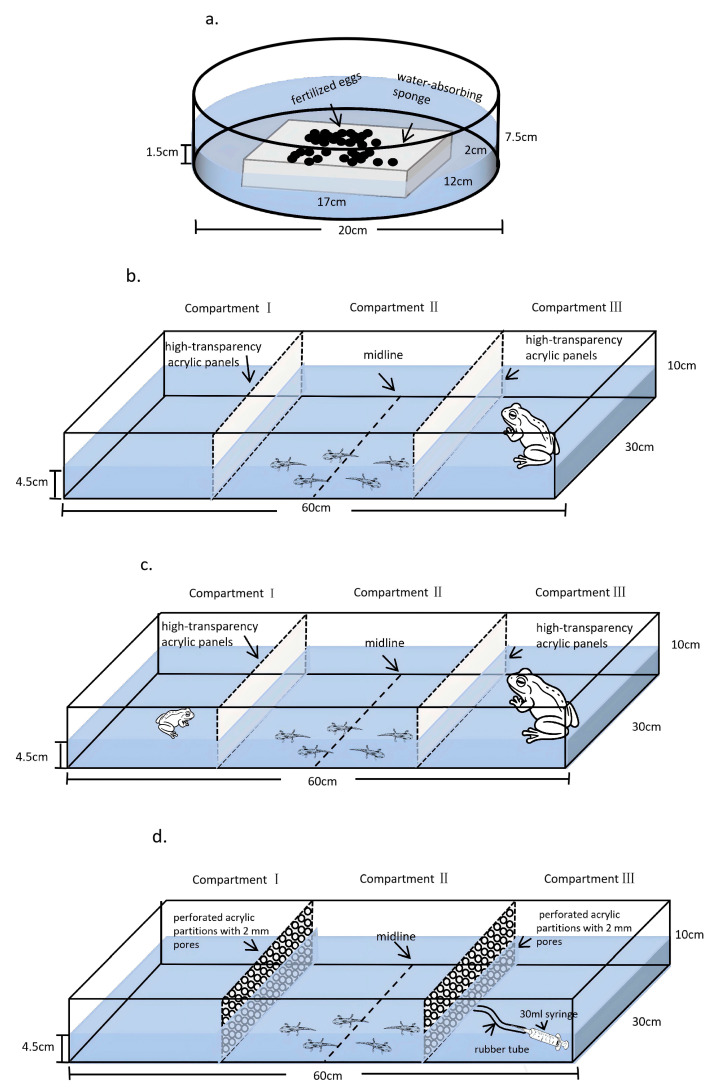
Schematic diagram of the different experimental setups: (**a**) incubation device; (**b**) visual cues alone; (**c**) visual cues from different size bullfrogs; (**d**) chemical cues alone.

We collected fertilized eggs of the Chinhai spiny newt from the Chinhai Spiny Newt Nature Conservation Area in Beilun District, Ningbo City, Zhejiang Province, China (29°48′24″ N, 121°51′12″ E). During the breeding season (April to May) of 2024. Then, the eggs were taken back to hatch under natural photoperiod and temperature conditions in our laboratory, within cylindrical plastic tanks (height: 7.5 cm, diameter: 20 cm) which was equipped with a 1.5 cm height of water and an ordinary water-absorbing sponge (17  × 12  ×  2 cm) placed within the water for each tank. That is, the eggs were hatched on the sponges, which remained in full contact with the water and maintained the humidity needed for egg hatching (Figure 1a). After the larvae hatched from the eggs, they were transferred to a plastic rearing box (45 × 29.5 × 14.5 cm) maintained with a water depth of 4.5 cm. Throughout the rearing period, the larvae were not exposed to any predators.

Large bullfrogs (weight: 258.26 ± 19.70 g) were purchased from Chaiqiao Fish Shop in Beilun District, Ningbo City, while small bullfrogs (weight: 13.93 ± 1.80 g) were obtained from Yizhiwa Aquatic Products Store in Chenghai District, Shantou City. In order to prevent infectious diseases and pollution in the following experiments, they were sterilized with potassium permanganate solution (1% in concentration), cleaned carefully with water, then reared in plastic boxes (see above) and fed locally collected frog tadpoles three times daily. After a 5-day acclimation period to ensure the stability of chemical cues, the bullfrogs were used for the experiments.

### 2.2. Experiment Design

#### 2.2.1. Experiment with Visual Cues Alone

The experimental setup consisted of a rectangular acrylic tank (60 × 30 × 10 cm). The outer sides of the tank were covered with opaque adhesive tape, and the interior was divided into three separate compartments using high-transparency acrylic panels (Figure 1b): Compartment I (15 × 30 cm), Compartment II (30 × 30 cm, where the experimental larvae were placed), and Compartment III (15 × 30 cm). A midline was marked on the bottom of Compartment II. Before the experiment, sun-exposed water was added to Compartments I and III, and the system was left undisturbed for 48 h to confirm that no leakage occurred through the dividers. Subsequently, the setup was drained, cleaned, and refilled with sun-exposed water to a height of 4.5 cm in all three compartments.

Five larvae were placed in Compartment II of the apparatus and allowed to acclimate to the experimental environment for 5 min. In the control experiments, no bullfrog individuals were placed in either Compartment I or Compartment III. The activity of larvae was recorded using a camera (Canon PowerShot SX60 HS, Canon Inc., Tokyo, Japan), and the number of larvae on the left and right sides of the midline in Compartment II was observed and recorded at different time points (2, 4, 6, 8, and 10 min). Following the control experiment, the same five larvae in the central compartment were retained for the subsequent visual recognition experiment. In the visual cues group, one bullfrog was randomly placed into either Compartment I or Compartment III. Video recording was conducted, and at each time point (2, 4, 6, 8, and 10 min), the number of larvae located in the distal zone relative to the bullfrog (i.e., the side of Compartment II separated by the midline that was farther from the bullfrog) was recorded. If a tadpole was positioned exactly on the midline at any given time point, it was assigned proportionally based on the relative body volume on each side of the midline. Additionally, the total number of times the five larvae crossed the midline within the 10 min experimental period was recorded as an indicator of their activity level. Both the control and visual cues groups were subjected to 30 repeated experiments, with different individual larvae used in each experiment.

#### 2.2.2. Experiment on Visual Cues of Different Body Sizes

Similarly to the aforementioned experiment (Figure 1c), five larvae were first introduced into the apparatus and allowed to acclimate for 5 min. Subsequently, either a large bullfrog or a small bullfrog was randomly placed in either Compartment I or Compartment III. Correspondingly, a small or large bullfrog was placed in the opposite compartment, such that both a large and a small bullfrog were present simultaneously in the same experiment, providing visual cues from predators of two distinct size classes. Video recording was conducted, and at each time point (2, 4, 6, 8, and 10 min), the number of larvae located in the distal zone relative to the large bullfrog and in the distal zone relative to the small bullfrog was recorded (with the midline of Compartment II as the boundary, the side farther from the large or small bullfrog was defined as the distal zone). A total of 30 repeated experiments were performed for this experiment, with different individual larvae used in each experiment.

#### 2.2.3. Experiment with Chemical Cues Alone

The experimental setup consisted of a rectangular acrylic tank (60 × 30 × 10 cm), divided into three compartments with the same dimensions as shown in Figure 1d. However, the compartments were separated by perforated acrylic partitions with a pore size of 2 mm, allowing water to flow between chambers while preventing larvae from passing through. Four healthy adult bullfrogs were kept in 1 L of sun-exposed water for 24 h and fed with frog tadpoles during this period. The chemical cues were prepared by filtering the water in which bullfrogs had been maintained, this conditioned water contained a mixture of bullfrog-derived metabolites, skin secretions, and other bioactive substances, thereby functioning as a composite chemical signal indicative of the recent presence of predators in the aquatic environment.

Before the experiment, the apparatus was thoroughly cleaned, placed on a level surface, and filled with sun-exposed water to a depth of approximately 4.5 cm. Five larvae were introduced into Compartment II of the apparatus and allowed to acclimate for 5 min. For the control group, 30 mL of plain sun-exposed water was evenly injected into either Compartment I or Compartment III. Throughout the experiment, video recording was used to document the number of larvae on the left and right sides of the midline in Compartment II at each time point (2, 4, 6, 8, and 10 min). After the control experiment, the same five larvae were retained, and 30 mL of chemical cue solution (water conditioned with bullfrog odor) was evenly injected randomly into one of the side compartments. The injection side was defined as the proximal end, and the opposite side as the distal end. Similarly, video was recorded, and the number of larvae located in the distal zone (away from the chemical cue) was recorded at each time point. The total number of midline crossings by the larvae within the 10 min period was also recorded as a measure of activity level. This concluded one experimental run. The apparatus was then cleaned again, refilled with fresh water, and five new larvae were introduced. The above steps were repeated for recording. Both the control and chemical cues groups were conducted with 30 replicate experiments each.

### 2.3. Data Analysis

First, the normality and homogeneity of variance for all data were assessed using the Shapiro–Wilk test. Normality of the data was assessed using the Shapiro–Wilk test. Since the data for “the number of tadpoles in the distal zone” consisted of repeated-measures discrete count data and did not meet the normality assumption, we used Generalized Estimating Equations (GEEs) for analysis [23]. This method effectively accounts for correlations among measurements taken at different time points from the same batch of larvae. Given that the data represented independent counts without overdispersion, the GEE model was specified with a Poisson distribution and a log-link function, and an exchangeable correlation structure was set to control for within-group correlations across repeated measurements. This analysis aimed to examine differences in “distal counts” across different treatment groups at five time points (2, 4, 6, 8, and 10 min). Data on “the total number of midline crossings by tadpoles” also did not follow a normal distribution; therefore, the non-parametric Wilcoxon signed-rank test was used for comparative analysis between groups.

All of the statistical analyses were processed in R 4.4.2 software [24]. The GEE was run in the geepack package (version 1.3.13) and the Wilcoxon signed-rank test was run in the stats package (version 4.4.2) in this study. Statistics here are shown as mean ± standard error (Mean ± SE) with a significance level at α = 0.05.

## 3. Results

### 3.1. Larvae Response to Visual Cues Alone

#### 3.1.1. Spatial Avoidance Behavior

**Figure 2:**
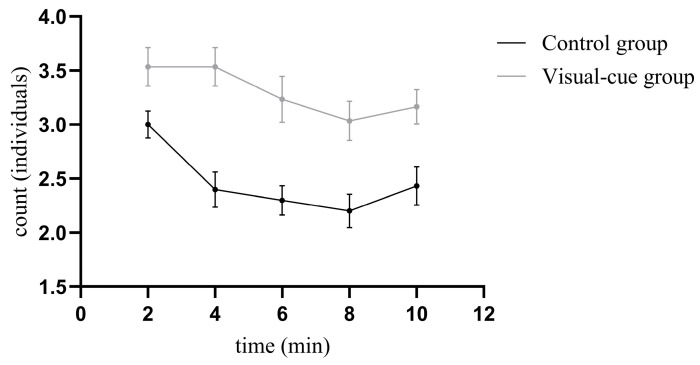
Trend in the number of larvae in the distal zone of the visual-cue group and the corresponding zone of the control group over time Data points were joined by a connecting line to illustrate the trend. Data points represent the mean values, and error bars indicate the Standard Error.

Under the condition of visual cues alone, the number of Chinhai spiny newt larvae located in the distal zone relative to the bullfrog showed a consistent temporal trend between the visual-cue groups and control groups (Figure 2): both declined from 0–8 min, followed by a slight rebound during 8–10 min. For the visual-cue group, over 60% of the larvae stayed away from the bullfrog throughout the 10 min experimental period (minimum = 3.033 ± 0.183 individuals, maximum = 3.533 ± 0.178 individuals). The highest number of larvae avoiding the bullfrog was observed within the first four minutes. In the control groups, the number of larvae in the distal zone peaked at 2 min and remained almost balanced during the 6–10 min period (maximum = 2.433 ± 0.177 individuals, minimum = 2.200 ± 0.155 individuals). The largest difference in the number of distal-zone larvae between the two groups occurred at 4 min (Table 1).

Based on the results of the Generalized Estimating Equation (GEE) model (Table 2), the between-group main effect was significant (β = 0.164, Wald χ^2^ = 6.416, *p* = 0.011). Indicating that the presence of bullfrog visual cues led to a significantly higher number of larvae in the distal zone in the visual-cue group compared to the control group. The time main effect was also significant: compared to the 2 min time point, the number of larvae in the distal zone decreased significantly at 4 min (β = −0.223, Wald χ^2^ = 11.442, *p* = 0.001), 6 min (β = −0.266, Wald χ^2^ = 14.617, *p* = 0.001), 8 min (β = −0.310, Wald χ^2^ = 18.655, *p* < 0.001), and 10 min (β = −0.209, Wald χ^2^ = 6.385, *p* = 0.011). A significant interaction effect between bullfrog stimulation and time was observed at 4 min (β = 0.182, Wald χ^2^ = 8.363, *p* = 0.018) and 6 min (β = 0.177, Wald χ^2^ = 4.167 *p* = 0.041). The positive interaction coefficients indicate that at these two time points, the decline in larval numbers in the visual-cue group was significantly less pronounced than in the control group, reflecting stronger and more sustained avoidance behavior toward the bullfrog during the mid-experimental period.

#### 3.1.2. Activity Level

**Figure 3:**
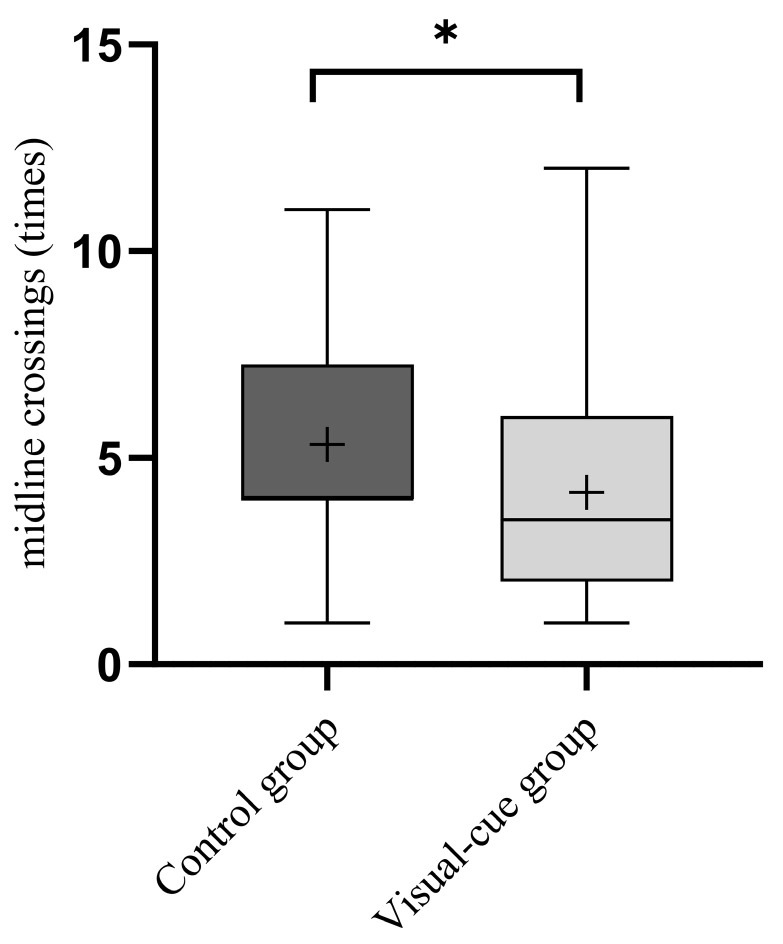
Wilcoxon signed-rank test on larval activity level under bullfrog visual cues. Box: Represents the interquartile range (IQR), spanning the 25th percentile (Q1) to the 75th percentile (Q3), indicating the central distribution range of the data. Error bars: Represent the maximum and minimum values within 1.5 × IQR, illustrating the data dispersion. Middle line: Represents the median of the data, reflecting the central tendency. Plus sign (+): Represents the mean of the data, distinguished from the median to visually show the average level of the data. The middle line of control group essentially coincides with the lower quartile (Q1) of the box plot. “*” indicates significant difference.

During the 10 min experimental period, the number of midline crossings by larvae in the visual-cue group (4.167 ± 2.718 times) was lower than that in the control group (5.333 ± 2.551 times). The results of the Wilcoxon Signed-Rank Test indicated that the presence of bullfrog visual cues significantly reduced the activity level of larvae (V = 288, *p* = 0.017; Figure 3).

### 3.2. Larvae Response to Chemical Cues Alone

#### 3.2.1. Spatial Avoidance Behavior

**Figure 4:**
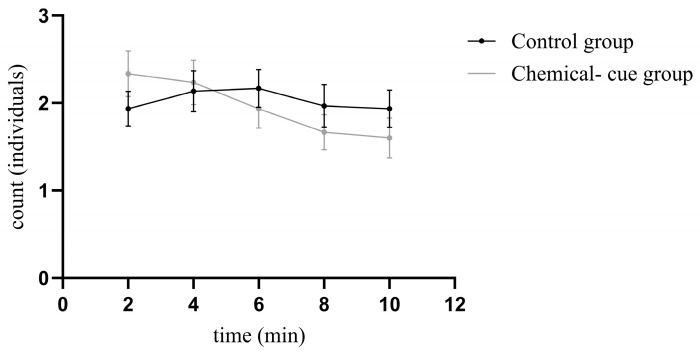
Trend in the number of larvae in the distal zone of the chemical-cue group and the corresponding zone of the control group over time Data points were joined by a connecting line to illustrate the trend. Data points represent the mean values, and error bars indicate the Standard Error.

The changes in the number of larvae avoiding the bullfrog-derived chemical cues and the corresponding number in the control group are shown in Figure 4 and Table 3. Larvae exhibited the highest number of individuals avoiding the chemical cues at 2 min (2.333 ± 0.260 individuals). This number decreased over time. In the control group, the number of larvae in the corresponding zone was lowest at 2 min (1.933 ± 0.197 individuals) and highest at 6 min (2.167 ± 0.215 individuals). From 6 to 10 min, the count in the corresponding zone of the control group was higher than the distal count in the chemical-cue group.

Based on the results of the Generalized Estimating Equation model (Table 4), the main effect of group was not significant (β = 0.188, Wald χ^2^ = 1.608, *p* = 0.205). This indicates no statistically significant difference in the number of larvae in the distal zone between the chemical-cue and control groups. Furthermore, for comparisons with the 2 min baseline, the time main effect was also not significant, meaning that the number of larvae in the control group remained stable throughout the experimental period. However, a significant e interaction effect between group and time was observed specifically at 6 min (β = −0.302, Wald χ^2^ = 4.117, *p* = 0.042), at this time point, the decrease in larval numbers in the chemical-cue group was significantly greater than that in the control group.

#### 3.2.2. Activity Level

**Figure 5:**
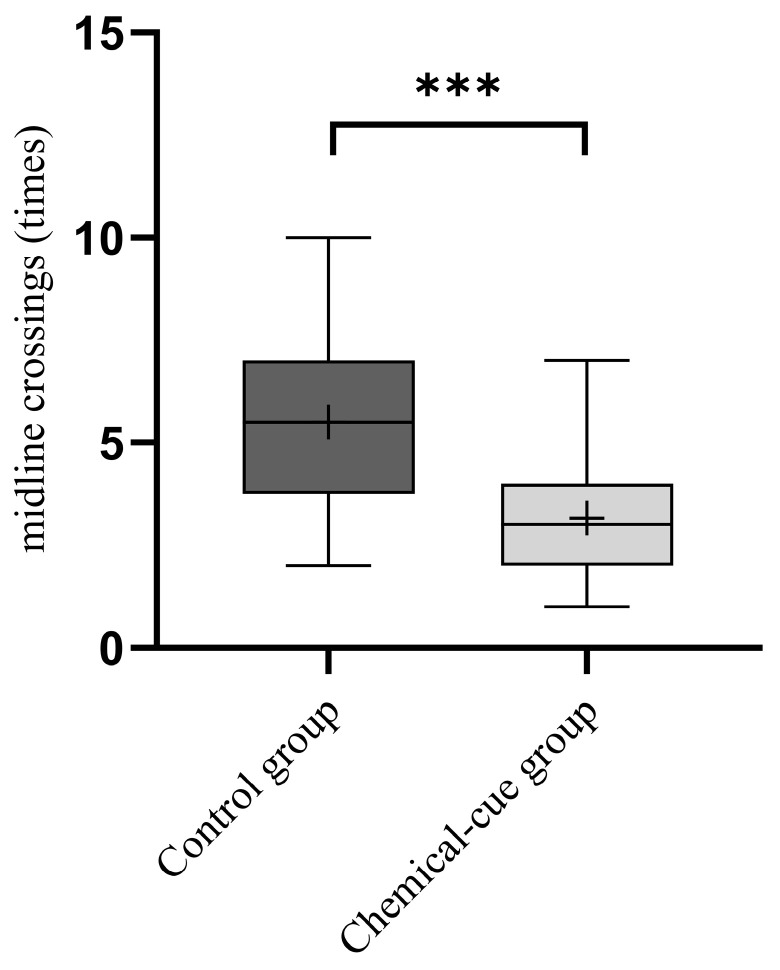
Wilcoxon signed-rank test on larval activity level under bullfrog chemical cues. “***” indicates significant difference.

In the experimental group, the total number of midline crossings by larvae within 10 min was 3.167 ± 1.555 times, which was significantly lower than that in the control group (5.500 ± 2.240 times). The results of the Wilcoxon Signed-Rank Test indicated that the chemical cues from bullfrogs affected the activity level of larvae. In an aqueous environment containing bullfrog predation chemical cues, the number of midline crossings by the larvae decreased very significantly (V = 314, *p* < 0.001, Figure 5).

### 3.3. Larvae Response to Visual Cues from Different Sizes of Bullfrogs

#### Spatial Avoidance Behavior

**Figure 6:**
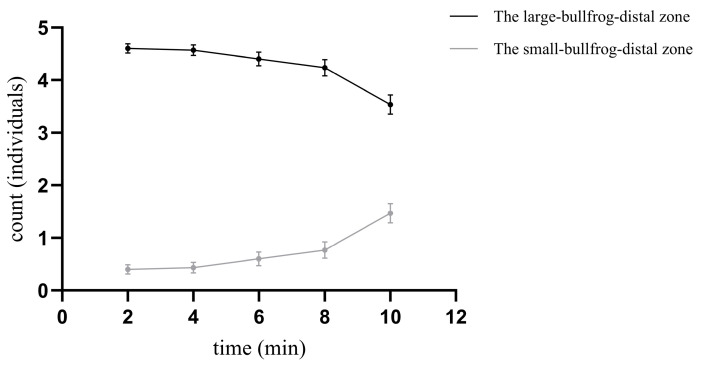
Trends in the number of larvae avoiding the large bullfrog and the small bullfrog.

When both large and small bullfrogs were simultaneously present, the changes in the number of larvae avoiding each stimulus side are shown in Figure 6 and Table 5. Throughout all time periods, the number of larvae avoiding the large bullfrog was consistently more than the number avoiding the small bullfrog (minimum for the large bullfrog side: 3.533 ± 0.184 individuals; maximum for the small bullfrog side: 1.467 ± 0.184 individuals). At 2 min, the number of larvae avoiding the large bullfrog reached its peak (4.600 ± 0.091 individuals). Regarding temporal trends, the two groups showed opposite patterns: the number of larvae on the large bullfrog side gradually decreased over time, while the number on the small bullfrog side gradually increased.

The results of the Generalized Estimating Equation model indicated that when visual stimuli from both large and small bullfrogs were present simultaneously, the larvae exhibited a clear preferential avoidance behavior (Table 6): the number of larvae in the large-bullfrog-distal zone was significantly more than that in the large-bullfrog-distal zone (β = 2.442, Wald χ^2^ = 118.406, *p* < 0.05). The time main effect showed that, with the distal zone relative to the small bullfrog at 2 min serving as the baseline, the number of larvae on the small-bullfrog-distal zone increased significantly at 8 min (β = 0.651, Wald χ^2^ = 5.004, *p* = 0.025) and 10 min (β = 1.299, Wald χ^2^ = 26.499, *p* < 0.001). Interaction effect analysis further revealed that the deterrent effect of the large bullfrog weakened significantly over time. Significant interaction effect was observed at 8 min (β = −0.734, Wald χ^2^ = 6.244, *p* = 0.012) and 10 min (β = −1.563, Wald χ^2^ = 36.658, *p* < 0.001), This indicates that at these two later time points, the decline in the number of larvae avoiding the large bullfrog was significantly more pronounced than in the earlier phase.

## 4. Discussion

### 4.1. The Primary Role of Visual Cues in Recognition

The results of this study indicate that the Chinhai spiny newt larvae are capable of recognizing the visual cues of novel predators. Their primary behavioral responses consist of spatial avoidance (moving away from the predator) and a reduction in activity level (decreased frequency of midline crossings). These responses were particularly pronounced during the early phase of visual exposure (0–6 min in the present experiment). Our findings support the role of visual cues in driving avoidance behavior in amphibians, which is consistent with the findings of Hettyey et al. [25]. However, some studies have indicated that visual cues in isolation might be limited and could fail to elicit a behavioral response from prey. For example, Stauffer and Semlitsch found in their study of American bullfrog (*Rana catesbeiana*) and green frog (*Rana clamitans*) tadpoles that exposure to visual predator cues alone did not elicit significant anti-predator behavior [26]. In this study, the considerable size difference between the bullfrog and the Chinhai spiny newt larvae, along with the clear experimental water, are both possible keys to the effective visual recognition. On the one hand, the distinct morphology and movement patterns of a large-bodied predator are likely more easily detected by the visual system of the larvae. On the other hand, the absence of vegetation in the experimental water further enhanced the reliability of visual signal transmission. Similarly, the importance of water clarity for visual predator recognition has been observed in other aquatic organisms. For instance, fathead minnows (*Pimephales promelas*) exhibit significantly reduced avoidance of hazardous areas in turbid water [27].

### 4.2. Warning Function of Chemical Cues

Our results indicate that under the sole influence of chemical cues from predators, the primary behavioral response of the Chinhai spiny newt larvae is a significant decrease in activity level. This indicates that the larvae are capable of responding to composite chemical cues we made. Similarly, this alertness behavior in response to predator chemical cues is well-documented across many organisms. For example, the eastern newt (*Notophthalmus viridescens*) exhibits a significant reduction in activity when exposed to chemical cues from the tiger salamander (*Ambystoma tigrinum*) [28]. Similarly, the San Marcos salamander (*Eurycea nana*), which has no prior experience with predators, also reduces its activity upon detecting chemical signals from non-native predatory fish [29]. We infer that certain components within the bullfrog chemical cues we provided may partially overlap with warning cues released by other native predators to the habitat of the Chinhai spiny newt. This could lead to a generalized predator recognition by the larvae [30], which leads to a significant decrease in activity. However, only the behavior of activity reduction was observed in our experiment, there will be relatively obvious avoidance behavior. We speculate a possible explanation that chemical cues are diffusive [6], and the space of the experimental device is limited. They disperse rapidly and distributed evenly in water, creating a pervasive signal of risk. Their primary function is to indicate the presence of danger rather than to pinpoint the predator’s exact location. Consequently, larvae may be unable to accurately determine the real-time position of the predator. In such a scenario, fleeing indiscriminately could increase the risk of encountering the undetectable threat. Therefore, reducing activity levels may be an optimal strategy. The Chinhai spiny newt larvae exhibit a generalized vigilance response to unfamiliar chemical signals, rather than a specific avoidance behavior. This reflects a conservative yet robust survival strategy adopted when confronting unknown environmental risks.

### 4.3. Risk Level Judgment Based on Body Size

This study demonstrates that when confronted with visual cues from bullfrogs of different body sizes, the Chinhai spiny newt larvae preferentially exhibit avoidance behavior toward the larger individual. This result may arise because animals can perceive key morphological features of predators—such as body size [31] and coloration [32]—through their visual system and subsequently translate these features into “risk-level signals” that trigger avoidance responses based on signal intensity [33,34]. In the present experiment, large bullfrogs were likely perceived by the larvae as high-risk signals, whereas small bullfrogs were registered as low-risk signals. When evaluating these two cues, the larvae exhibited a clear preference for remaining closer to small bullfrogs while actively avoiding large ones. This behavior indicates that the larvae were capable of conducting risk assessments within the confined experimental space and selecting the less perilous option. Our results extend the application of threat-sensitive theory, which posits that prey adjust their behavioral responses according to perceived threat levels [35]. The present study further suggests that when faced with multiple risk sources, prey not only react but can also access and choose between risks. Similar patterns of risk assessment have been observed in other taxa and species. For instance, zebrafish (*Danio rerio*) exhibit faster escape responses when confronted with larger predators such as the Japanese seabass (*Lateolabrax japonicus*) [36]. Similarly, Thomson’s gazelle (*Eudorcas thomsonii*) displays increased flight speed when encountering larger-bodied predators [37].

## 5. Conclusions

This study investigated the recognition mechanisms and behavioral responses of the Chinhai spiny newt larvae to a novel predator through controlled experiments involving visual cues alone, chemical cues alone, and visual cues from predators of different body sizes. The results indicated that the larvae could perceive predator threats via visual or chemical signals and exhibit adaptive antipredator behaviors, such as avoidance or activity reduction. Additionally, they could assess differences in predator body size based on visual information and, accordingly, differentiate and weigh risk levels. These behavioral responses represent the larvae’s reaction to novel potential predators. Notwithstanding these findings, it is important to acknowledge limitation of the present study that we only focused on the roles of visual and chemical cues, without incorporating acoustic signals into the experimental design. In the future, we could adopt a multisensory signal integration experimental design that incorporates acoustic, visual, and chemical cues to comprehensively analyze the risk assessment mechanism of the larvae.

Similarly, while this study confirms anti-predator responses in *E. chinhaiensis* larvae towards novel predators, future research must incorporate native predator controls. Directly comparing larval behavioral responses to different predator cues is essential to establish whether recognition is specific to invasive species and to elucidate the underlying behavioral mechanisms. This is vital for a full understanding of the species’ behavioral plasticity and survival prospects in complex predatory landscapes.

## Figures and Tables

**Table 1:** The count of larvae located in the distal zone relative to the bullfrog, in both the visual-cue group and the control group at each time point.

Measurement Indicators	Time (min)	Control Group (*n* = 30, Mean ± SE)	Visual-Cue Group (*n* = 30, Mean ± SE)
Distal tadpole count	2	3.000 ± 0.127	3.533 ± 0.178
	4	2.400 ± 0.163	3.533 ± 0.178
	6	2.300 ± 0.137	3.233 ± 0.213
	8	2.200 ± 0.155	3.033 ± 0.183
	10	2.433 ± 0.177	3.167 ± 0.160

**Table 2:** Results of the Generalized Estimating Equation model for the visual cues in the control and visual-cue groups (*n* = 30). Significant differences (*p* < 0.05) between different groups are highlighted in bold.

	β (Estimate)	SE	Wald χ^2^	*p*-Value
(Intercept)	1.099	0.042	698.306	**<0.001**
Group	0.164	0.065	6.416	**0.011**
time 4 vs. 2	−0.223	0.066	11.442	**0.001**
time 6 vs. 2	−0.266	0.069	14.617	**<0.001**
time 8 vs. 2	−0.310	0.072	18.655	**<0.001**
time 10 vs. 2	−0.209	0.083	6.385	**0.012**
group × time4	0.233	0.077	8.363	**0.004**
group × time6	0.177	0.087	4.167	**0.041**
group × time8	0.158	0.097	2.657	0.103
group × time10	0.100	0.106	0.881	0.348

**Table 3:** The count of larvae located in the distal zone in both the chemical-cue group and the control group at each time point.

Measurement Indicators	Time (min)	Control Group (*n* = 30, Mean ± SE)	Chemical-Cue Group (*n* = 30, Mean ± SE)
Distal tadpole count	2	1.933 ± 0.197	2.333 ± 0.260
	4	2.133 ± 0.234	2.233 ± 0.252
	6	2.167 ± 0.215	1.933 ± 0.219
	8	1.967 ± 0.242	1.667 ± 0.200
	10	1.933 ± 0.214	1.600 ± 0.228

**Table 4:** Results of the Generalized Estimating Equation Model for changes in the number of larvae away from chemical cues in the control and chemical-cue groups under the effect of chemical cues (*n* = 30). Significant differences (*p* < 0.05) between different groups are highlighted in bold.

	β (Estimate)	SE	Wald χ^2^	*p*-Value
Intercept	0.659	0.100	43.170	**<0.001**
Group	0.188	0.148	1.605	0.205
time 4 vs. 2	0.098	0.087	1.291	0.256
time 6 vs. 2	0.114	0.102	1.251	0.263
time 8 vs. 2	0.017	0.146	0.014	0.907
time 10 vs. 2	0.000	0.152	0.000	1.000
group × time4	−0.142	0.120	1.398	0.237
group × time6	−0.302	0.149	4.117	**0.042**
group × time8	−0.354	0.211	2.803	0.094
group × time10	−0.377	0.233	2.627	0.105

**Table 5:** The number of larvae in the distal zone relative to the large bullfrog and the small bullfrog at each time point.

Measurement Indicators	Time (min)	The Small-Bullfrog-Distal Zone (*n* = 30, Mean ± SE)	The Large-Bullfrog-Distal Zone (*n* = 30, Mean ± SE)
Distal tadpole count	2	0.400 ± 0.0910	4.600 ± 0.091
	4	0.433 ± 0.1038	4.567 ± 0.104
	6	0.600 ± 0.1322	4.400 ± 0.132
	8	0.767 ± 0.1567	4.233 ± 0.157
	10	1.467 ± 0.1840	3.533 ± 0.184

**Table 6:** Results of the Generalized Estimating Equation Model for changes in the number of larvae away from the small-bullfrog-distal zone and the large-bullfrog-distal zone (*n* = 30). Significant differences (*p* < 0.05) between different groups are highlighted in bold.

	β (Estimate)	SE	Wald χ^2^	*p*-Value
Intercept	−0.916	0.224	16.792	**<0.001**
Group	2.442	0.224	118.406	**<0.001**
time 4 vs. 2	0.080	0.308	0.067	0.795
time 6 vs. 2	0.405	0.283	2. 049	0.152
time 8 vs. 2	0.651	0.291	5.004	**0.025**
time 10 vs. 2	1.299	0.252	26.499	**<0.001**
group × time4	−0.087	0.310	0.079	0.778
group × time6	−0.450	0.285	2.490	0.115
group × time8	−0.734	0.294	6.244	**0.012**
group × time10	−1.563	0.258	36.658	**<0.001**

## Data Availability

Data will be made available on demand.
